# Battery-SOC Estimation for Hybrid-Power UAVs Using Fast-OCV Curve with Unscented Kalman Filters

**DOI:** 10.3390/s23146429

**Published:** 2023-07-15

**Authors:** Zhuoyao He, David Martín Gómez, Arturo de la Escalera Hueso, Pablo Flores Peña, Xingcai Lu, José María Armingol Moreno

**Affiliations:** 1 Intelligent Systems Laboratory (LSI), Universidad Carlos III de Madrid, Av. Universidad 30, Leganés, 28911 Madrid, Spain; escalera@ing.uc3m.es (A.d.l.E.H.); armingol@ing.uc3m.es (J.M.A.M.); 2 Key Laboratory for Power machinery and Engineering of M.O.E., Shanghai Jiao Tong University, Dongchuan Road No. 800, Shanghai 200240, China; lyuxc@sjtu.edu.cn; 3 Drone Hopper S.L., Avenida Gregorio Peces-Barba, Leganés, 28919 Madrid, Spain; pablo.flores@drone-hopper.com

**Keywords:** open-circuit voltage, state-of-charge estimation, battery-parameter identification, unscented Kalman filter, hybrid power, unmanned aerial vehicles

## Abstract

Unmanned aerial vehicles (UAVs) have drawin increasing attention in recent years, and they are widely applied. Nevertheless, they are generally limited by poor flight endurance because of the limited energy density of their batteries. A robust power supply is indispensable for advanced UAVs; thus hybrid power might be a promising solution. State of charge (SOC) estimation is essential for the power systems of UAVs. The limitations of accurate SOC estimation can be partly ascribed to the inaccuracy of open circuit voltage (OCV), which is obtained through specific forms of identification. Considering the actual operation of a battery under hybrid conditions, this paper proposes a novel method, “fast OCV”, for obtaining the OCVs of batteries. It is proven that fast OCV offers great advantages, related to its simplicity, duration and cost, over traditional ways of obtaining OCV. Moreover, fast-OCV also shows better accuracy in SOC estimation than traditional OCV. Furthermore, this paper also proposes a new method, “batch mode”, for talking-data sampling for battery-parameter identification with the limited-memory recursive least-square algorithm. Compared with traditional the “single mode”, it presents good de-noising effect by making use of all the sampled battery’s terminal current and voltage data.

## 1. Introduction

As flying robots, UAVs are receiving increasing attention, with the advancement of microprocessor and artificial technologies [1]. Due to their advantages, such as their low cost and high mobility, UAVs are widely applied in numerous activities, like rescuing, monitoring, delivery, agriculture, and even military investigations, as well as battlefields [2,3,4], etc. However, the performances of UAVs are closely related to on-board power capabilities [5]. Generally, batteries are used as the main sources of power for UAVs; hence, the accurate assessment of the power states of batteries is extremely important for UAV missions [6,7].

The SOC, which means the charge available relative to the full charge capacity of a battery, is generally taken as a vital indicator for battery management [8,9]. Failures in SOC estimation can potentially lead to overcharging, over-discharging, or even irreversible damage to batteries [10]. However, the direct measurement of the SOC of a battery is not possible. In practical applications, indirect approaches are usually adopted for SOC estimation. Nevertheless, an accurate assessment of the SOC is usually of highly challenging, as it is complicated and associated with many factors [7,11], such as the charge-and-discharge efficiency, the charge-and-discharge rate, the temperature [12], etc. Thus far, various methods, like coulomb counting [13], artificial intelligence [14,15], the fuzzy logic algorithm, and Kalman filters [16,17,18] have been widely investigated and used in SOC estimation. In comparison, the model-based method is relatively popular due to its simplicity [7,11]. Specifically, equivalent circuit models (ECM) combined with Kalman filters are the most popular, since they are widely adopted by numerous researchers.

As a function of SOC, the open-circuit voltage (OCV) is crucial for SOC estimation. It is usually used as a reference for innovation, which helps to improve the accuracy of state estimation for Kalman-filter-based SOC estimation. The crucial influence of the OCV on SOC estimation was disclosed in related studied through a large number of ECMs [19,20]. Currently, incremental OCV testing and low-current testing are usually used for OCV-curve abstraction [21,22]. However, such methods are usually time-consuming, owing to the battery-relaxation process. Furthermore, the complete measurement of OCV curves is not exclusive, as changes occur due to temperature and the battery’s rate of aging [23,24,25]. Moreover, different batteries do not share the same OCV curve, even though they are from the same production line. All these factors indicate the strong necessity of efficient ways of obtaining OCV curves for batteries in practical applications.

To address these issues, several studies have been conducted by numerous researchers. Zhang et al. [26] proposed a non-experiment-based OCV-reconstruction method. However, it was mainly limited to a partial updating of an OCV curve. Fan et al. [27] introduced a method for efficiently reconstructing an OCV curve while considering the temperature variation of a battery by inspecting its charge-and-discharge process. Similarly, Cui et al. [28] conducted an OCV-curve reconstruction based on an electrode-OCV model; the results proved its effectiveness under different temperature conditions. However, these methods involved complex processed and struggled to meet time-efficiency requirements. Some other relevant studies were presented by many researchers [29,30,31,32] in recent years. Despite their significant achievements, these studied usually tried to establish possible ways of obtaining OCV curves as universally applicable. However, these methods do not offer positive solutions as specific conditions were not considered.

According to the idea of configuration, hybrid power systems that assemble engine/fuel cells and batteries can be classified as serial, parallel, or serial-parallel [33,34,35]. Regardless of the type, however, the engine/fuel cell is usually the primary power source, while the battery is an auxiliary part [36,37]. Regarding the application of hybrid power in UAVs, the battery can be better configured when it has a small capacity and, hence, light weight, as UAVs are weight-sensitive. Importantly, the ability to control charging and discharging of the battery can be achieved through the hybrid-powered UAV system, which is very similar to the core function of the professional battery-testing system. With this consideration, this paper aims to propose a fast and simple method for obtaining battery-OCV curves by making use of the configuration of the whole system. The obtained OCV curve is validated by conducting a SOC estimation for a battery with Kalman filters under different working conditions.

As previously stated, Kalman filters (KFs), extended Kalman filter (EKFs), and unscented Kalman filters (UKFs), are the most popular approaches to SOC estimation. In terms of comparison, UKFs are drastically superior to KFs and EKFs when tackling non-linear systems due to their unscented transformation [38] and, hence, good robustness and high accuracy [39,40]. He et al. [41] conducted a comparative investigation of EKFs and UKFs for SOC estimation. The results showed that the UKF provided much better predictions. Zhang et al. [42] investigated a UKF for SOC estimation, and good predictions were obtained. Nevertheless, Kalman filters entail the predetermined covariance of the state and observation variables. However, these parameters are usually unknown and mainly set according to experiences. To overcome this drawback, in recent years, researchers have tended to adopt the Sage–Husa noise estimator for the online estimation of the covariance mentioned previously [43,44]. Combined with the noise estimator, an adaptive UKF (AUKF) was presented. Therefore, this paper attempts to validate an OCV curve obtained through battery-SOC estimations with UKF and AUKF.

The structure of this paper is as follows. In Section 2, we first discuss the ECM related to this work, and then the method of efficiently obtaining the OCV curve on the UAV with a hybrid power system is introduced theoretically and practically. In Section 3, the dynamic battery model and the AUKF specifically used in this research are discussed. In Section 4, the experiments involved in and the results of the method’s validation are presented. Lastly, we draw conclusions in Section 5, in which we also discuss our expectations regarding future studies.

## 2. Model Selection, Testing Method, and Parameter Identification

### 2.1. Equivalent Circuit Models

**Figure 1:**
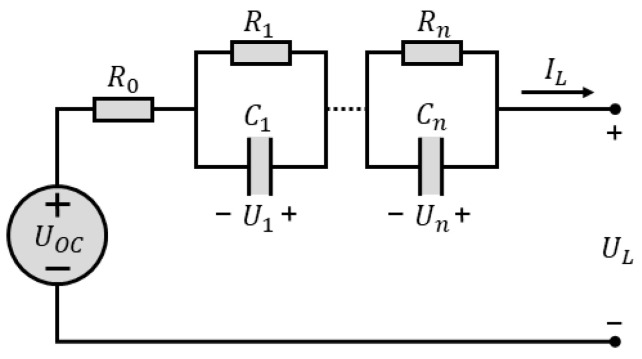
Schematic diagram of n-order polarization model.

To conduct a precise evaluation of battery characteristics, a robust model that describes the battery is essential. Numerous researchers have developed various models, which can generally be categorized into electrochemical models, neuro-network models, and equivalent circuit models (ECMs), among which ECMs are the most widely used owing to their simplicity and clear physical significance. In this paper, an ECM with n RC networks, which is called the n-order-polarization model, shown in Figure 1, is considered. Case n equals 1 (Thevenin model), is thoroughly involved in this investigation. However, case n equals 2 (dual polarization (DP) model) was also referred to during the model selection, while n equals 0 (Rint model) was used when obtaining the OCV.

With the advantage of calculation simplicity, Rint model is mainly applicable in situations with large currents, and transient characteristics are not considered. Thevenin model is constructed by using a parallel RC, which mimics the polarization effect of battery, in Rint model. Therefore, transient characteristics can be simulated. By introducing another parallel RC, which describes the diffusion effect of battery, to Thevenin mode, DP model is created. This model is usually able to describe the dynamic performance of a battery accurately.

### 2.2. Open-Circuit Voltage and Testing Method

For a precise SOC estimation, an OCV with relatively good accuracy is indispensable. Ordinarily, OCV can be observed by direct measurement after a sufficiently long time of rest for the battery. This method requires a significant amount of time to complete OCV observation. Many researchers conducted numerous investigations aiming at proposing potential universally applicable solutions. Examples include data-driven and model-driven solutions [31]. These methods may not always work when time efficiency and specific scenarios are considered. For battery in hybrid mode, online charging and discharging are naturally available. Considering the simplicity and applicability of OCV-construction method, specifically for battery in hybrid mode, this paper attempts to propose a simple method for OCV observation, as explained in the following content.

**Figure 2:**
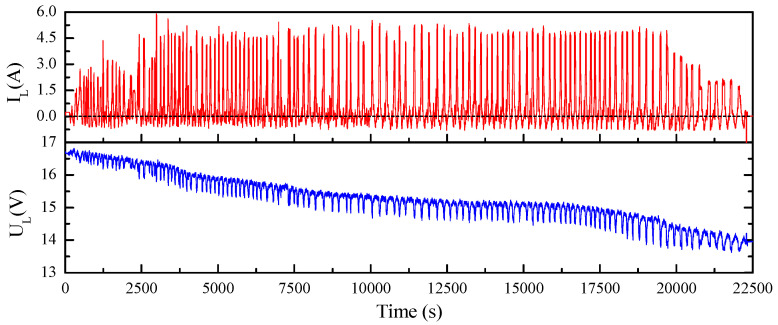
Charging-and-discharging process adopted in this paper.

Take the simplest ECM, Rint model, into consideration and consider polynomial description of $R_{0}$ and OCV versus SOC, as shown in Equations (1) and (2). According to Figure 1, relationship between output voltage $U_{L}$ and OCV can be formulated as Equation (3). The OCV equals $U_{L}$ if $I_{L}$ is set as 0; thus, OCV can be obtained if the coefficients in Equations (1) and (2) are solved. In fact, this can be easily achieved through least-square method by collecting the charging/discharging data of the battery. For a better solution, charging and discharging were randomly shifted with a global discharging process for the test battery, which was initially fully charged. The current and voltage curves for the process mentioned here are shown in Figure 2.

(1) $$R_{0}=a_{0}+a_{1}SOC+\ldots +a_{n}{SOC}^{n}$$

(2) $$OCV=b_{0}+b_{1}SOC+\ldots +b_{n}{SOC}^{n}$$

(3) $$U_{L}=OCV-I_{L}R_{0}$$

**Figure 3:**
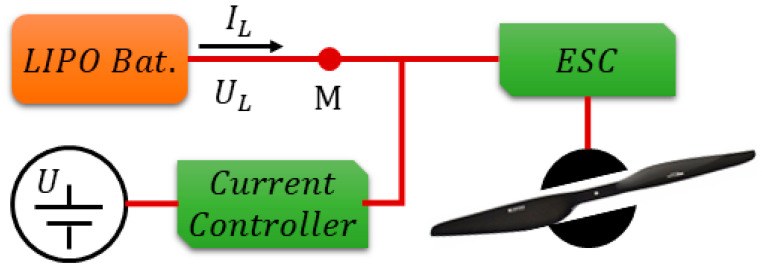
The method for realizing charging and discharging adopted in this paper.

However, to realize this process, as well as other tests, which are discussed below, a test set was created, as depicted in Figure 3. Battery was connected to motor propellers of UAV, while another power source with current controller was connected in parallel. Measurements of $U_{L}$ and $I_{L}$ focused on battery at point M. When setting current from fixed power-current controller, variations in the rotating speed of the motor propellers led to the variation of $I_{L}$ in the battery.

**Figure 4:**
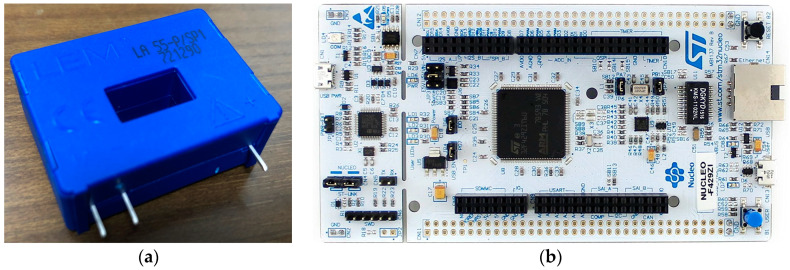
The key facilities used for signal sensing, testing-system control, and data sampling: (**a**) current transducer LA 55-P/SP1 used for current sensing; (**b**) STM32 Nucleo-144 boards F429ZI (MB1137) used for voltage sensing, data sampling, and system control.

**Figure 5:**
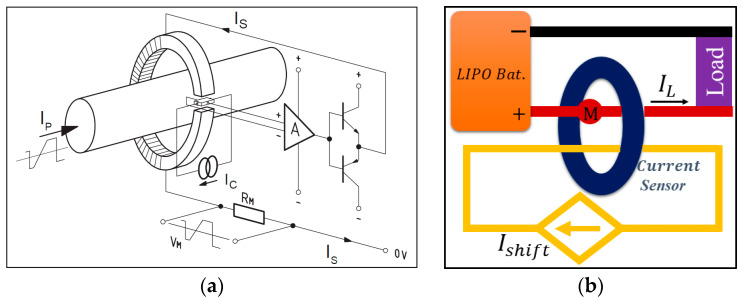
The method for sensing current reported in this paper: (**a**) operating principle of current transducer LA 55-P/SP1; (**b**) the method of current measurement with shift current in this paper.

For accurate current sensing, product LA 55-P/SP1, shown in Figure 4a, from LEM (Life Energy Motion) company was used. The principle of this transducer is shown in Figure 5a. When the primary current $I_{p}$ (the target for measuring) passes through the coil, induced magnetic field is sensed by the Hall generator driven by current $I_{c}$ with output of Hall voltage. This voltage is further sensed by closed-loop sensing circuit, which is mainly composed of an operational amplifier and a potential clamp. This closed-loop sensing circuit senses Hall voltage and drives the secondary current $I_{s}$ to compensate the Hall voltage as zero. By sensing the voltage drop $V_{M}$ on $R_{M}$, $I_{p}$ is yielded. The $I_{p}$ is forward when $V_{M}$ is positive, and vice versa. The key parameters and measuring accuracy are shown in Table 1, Table 2 and Table 3.

For controlling needs, STM32 Nucleo-144 boards, F429ZI (MB1137), were used to generate the driving signal fed to ESC. The Soc-STM32F429-embedded 12-bit ADC converter was mused to measure all the voltage signals. However, this ADC converter was not capable of converting negative voltage. Therefore, a constant current $I_{shift}$ was fed across the current transducer, driving working state of the transducer to roughly the middle of positive measuring span when $I_{L}$ equalled zero. By subtracting $I_{shift}$ from the lumped measurement, $I_{L}$ (either positive or negative) was yielded.

The sensing span of ADC converter on STM32F429 was between 0 V and 3 V. To measure the voltage signals exceeding this range, voltage-divider circuit composed of resister was applied for signal conversion. However, considering the accuracy of the circuit, RS PRO Mini AC/DC Clamp (Stock No. 146-9096) was used for calibration of voltage-signal measurement.

**Figure 6:**
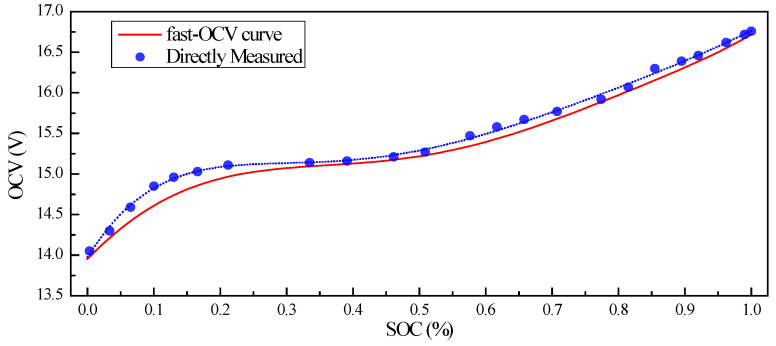
OCV obtained through direct measurement and dynamic current experiments (fast OCV) described in this paper.

In this paper, 6-order polynomial was adopted for Equations (1) and (2). Basic information on the battery and the coefficients solved is shown in Table 4. For comparison, OCV derived through traditional measurement is also included in this paper, as shown in Figure 6.

### 2.3. Parameter Identification

In this paper, we consider Thevenin model and DP model for battery description. However, before SOC estimation, $R_{i}$ and $C_{i}$ in the model should be identified. This paper uses limited-memory recursive least-square algorithm to achieve parameter identification.

Consider DP model of battery. Time-related relationship between $U_{OC}(t)$, $I_{L}(t)$, $U_{1}(t)$ and $U_{2}(t)$ can be described with Equations (4)–(6).

(4) $$U_{OC}(t)=I_{L}(t)R_{0}+U_{1}(t)+U_{2}(t)+U_{L}(t)$$

(5) $$I_{L}(t)=U_{1}(t)/R_{1}+C_{1}{dU}_{1}(t)/dt$$

(6) $$I_{L}(t)=U_{2}(t)/R_{2}+C_{2}{dU}_{2}(t)/dt$$

After Laplace transformation, Equations (7)–(9) can be obtained.

(7) $$U_{OC}(s)=I_{L}(s)R_{0}(s)+U_{1}(s)+U_{2}(s)+U_{L}(s)$$

(8) $$I_{L}(s)=U_{1}(s)/R_{1}+C_{1}sU_{1}(s)$$

(9) $$I_{L}(s)=U_{2}(s)/R_{2}+C_{2}sU_{2}(s)$$

With a summary of Equations (7)–(9), the relationship between $U_{OC}(t)$, $I_{L}(t)$, $U_{1}(t)$ and $U_{2}(t)$ can be described by Equation (10).

The definitions of a, b, c, *d*, and e are shown in Equation set (11), where $\tau_{1}$ and $\tau_{2}$ signify $R_{1}C_{1}$ and $R_{2}C_{2}$.

(10) $$\left(1+as+bs^{2}\right)U_{OC}(s)=\left(c+es+ds^{2}\right)I_{L}(s)+(1+as+bs^{2})U_{L}(s)$$

(11) $$\left\{\begin{array}{l} a=\tau_{1}+\tau_{2}\\ b=\tau_{1}\tau_{2}\\ c=R_{0}+R_{1}+R_{2}\\ d=\tau_{1}\tau_{2}R_{0}\\ e=\left(\tau_{1}+\tau_{2}\right)R_{0}+\tau_{1}R_{2}+\tau_{2}R_{1} \end{array}\right.$$

By identifying a, b, c, *d*, and e in Equation set (11), parameters $R_{0}$, $R_{1}$, $C_{1}$, $R_{2}$, and $C_{2}$ can be determined. To achieve this, Equation (10) is transformed into (12) through inverse Laplace transformation.

(12) $$U_{OC}\left(t\right)+a\frac{{dU}_{OC}\left(t\right)}{dt}+b\frac{{d^{2}U}_{OC}\left(t\right)}{dt^{2}}={cI}_{L}\left(t\right)+d\frac{{dI}_{L}\left(t\right)}{dt}+e\frac{{d^{2}I}_{L}\left(t\right)}{dt^{2}}+U_{L}+a\frac{{dU}_{L}\left(t\right)}{dt}+b\frac{{d^{2}U}_{L}\left(t\right)}{dt^{2}}$$

Discretization can then be applied to (12). In this investigation, the current from the battery is small in relation to relatively high capacity of the battery; thus derivative terms ${dU}_{OC}\left(t\right)/dt$ and ${d^{2}U}_{OC}\left(t\right)/dt^{2}$ are negligible and, hence, ignored.

(13) $$w_{0}+w_{1}I_{t}+w_{2}I_{t-T}+w_{3}I_{t-2T}+w_{4}U_{L,t-T}+w_{5}U_{L,t-2T}=U_{L,t}$$

(14) $$\left\{\begin{array}{l} w_{0}=T^{2}U_{OC,t}/M\\ w_{1}=-(cT^{2}+eT+d)/M\\ w_{2}=(eT+2d)/M\\ w_{3}=-d/M\\ w_{4}=(aT+2b)/M\\ w_{5}=-b/M \end{array}\right.$$

Let $M=T^{2}+aT+b$, where T is the refresh-time interval. The discretized formation of (12) is obtained as (13). Coefficients $w_{0}\sim w_{5}$ can be expressed by coefficients a~e through Equation set (14). By solving Equation (13), $w_{0}\sim w_{5}$ can be determined, and a~e, $R_{0}$ $R_{1}$, $C_{1}$, and $R_{2}$, and $C_{2}$ can be obtained.

(15) $$X_{t,i}=(1,I_{t-ih},I_{t-T-ih},I_{t-2T-ih},U_{L,t-T-ih},U_{L,t-2T-ih})$$

(16) $$Y_{t,i}=(U_{L,t-ih})$$

(17) $$W_{t}={\left(w_{0,t},w_{1,t},w_{2,t},w_{3,t},w_{4,t},w_{5,t}\right)}^{T}$$

(18) $$\left\{\begin{array}{l} K_{t}=P_{t-T}X_{t}^{T}{\left(\alpha E+X_{t}P_{t-T}X_{t}^{T}\right)}^{-1}\\ P_{t}=\left(E-K_{t}X_{t}\right)P_{t-T}/\alpha \\ W_{t}=W_{t-1}+K_{t}\left(Y_{t}-X_{t}W_{t-1}\right) \end{array}\right.$$

(19) $$P_{0}={\left(X_{0}^{T}X\right)}^{-1}$$

(20) $$W_{0}=P_{0}X_{0}^{T}Y_{0}$$

In this paper, limited-memory recursive least squares is used to solve the equation to avoid data saturation. The α is set as 0.99. To initiate the algorithm, a set of data are used to give reasonable initial value for P and W through Equations (19) and (20).

All above are related to DP model. However, the deduction of algorithm for Thevenin model is very similar; hence, it is omitted for simplicity.

**Figure 7:**
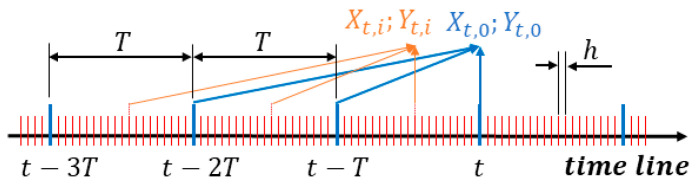
Explanation of $X_{t,i}$ $Y_{t,i}$; sample time h and refresh time T.

The method for creating $X_{t}$ and $Y_{t}$ is shown in Figure 7. In the experiments, sample time h was 0.064 s, and a calculation was conducted every 20 times on each sample, i.e., refresh time T = 20 × h = 1.28 s; thus, the estimated parameters were refreshed. In fact, limited-memory recursive least squares is mainly used for online calculations. Thus, $X_{t}$ usually only contains one sample to precipitate the calculation process. We call it “single mode” here. In contrast, case $X_{t}$ contains more than one sample. Here, it is called “batch mode”. The $X_{t}$ contains 20 samples with batch mode in this investigation.

**Figure 8:**
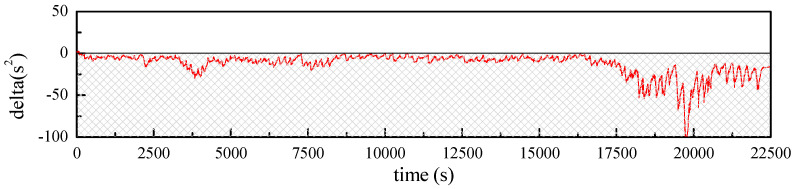
Values of delta during the entire test.

For DP model, parameter estimation involves solving quadratic equation. Specifically, $delta=a^{2}-4b$ should be non-negative; thus a real solution for the parameters can be realized. However, results proved that delta value for the test was always negative, which means that DP model is not suitable for the battery in this research. Thus, Thevenin model is adopted in the following discussion (Figure 8).

**Figure 9:**
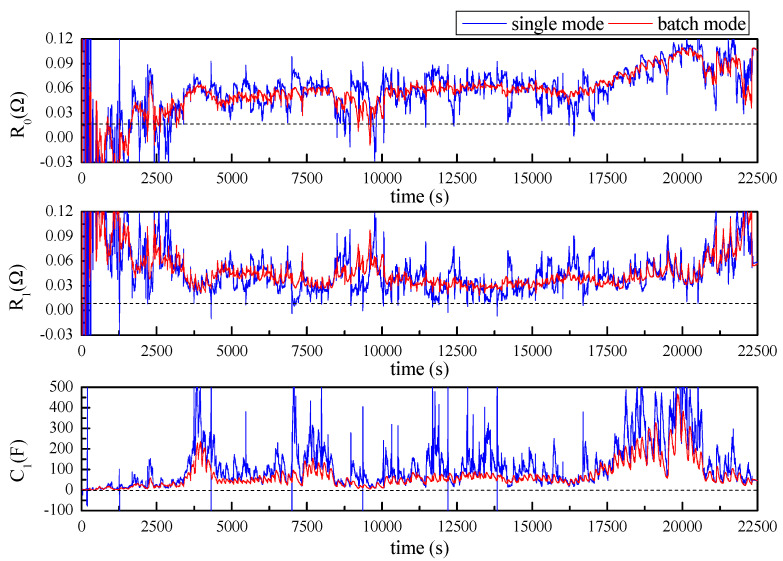
Estimated parameters $R_{0}$, $R_{1}$, and $C_{1}$ with Thevenin model.

As shown in Figure 9, parameters $R_{0}$, $R_{1}$, and $C_{1}$ are successfully estimated and batch mode is obviously superior to single mode in terms of prediction stability. By tracking SOC of the battery, a lookup table of $R_{0}$, $R_{1}$, and $C_{1}$ versus SOC was developed for SOC estimation under working conditions.

## 3. AUKF for SOC Estimation

### 3.1. State Equation and Observation Equation for Thevenin Battery Model

According to the Thevenin model, it is obvious that the $U_{OC}$ or SOC and $U_{1}$ can be selected as the complete state variables for the battery. For convenience, we consider SOC instead of $U_{OC}$ to avoid a conversion from $U_{OC}$ to SOC when making predictions. Therefore, the state-transfer equation can be expressed as (23), where $\varepsilon_{t}$ is the process-noise vector.

(21) $${SOC}_{t}={SOC}_{0}-\int_{0}^{t}\eta I_{\tau }d\tau /C_{a}$$

(22) $$U_{1,t}=(1-T/\tau_{1})U_{1,t-T}+TI_{t}/C_{1}$$

(23) $$\left[\begin{array}{c} {SOC}_{t}\\ U_{1,t} \end{array}\right]=\left[\begin{array}{cc} 1 & 0\\ 0 & 1-\frac{T}{\tau_{1}} \end{array}\right]\left[\begin{array}{c} {SOC}_{t-T}\\ U_{1,t-T} \end{array}\right]+\left[\begin{array}{c} -\frac{\eta T}{C_{a}}\\ \frac{T}{C_{1}} \end{array}\right]I_{t}+\varepsilon_{t}$$

The ${SOC}_{t}$ value from the initial value of the ${SOC}_{0}$ by the actuation of current $I_{t}$ can be expressed as Equation (21), where η is the charging efficiency and $C_{a}$ is the capacity of the battery. The effect of the actuation $I_{t}$ on $U_{1}$ can be expressed as Equation (22). Therefore, the state-transfer equation can be expressed as

(24) $$U_{L,t}=U_{OC}\left({SOC}_{t}\right)-U_{1,t}-R_{0}I_{t}+\delta_{t}$$

Similarly, the observation equation can be expressed as (24), where $\delta_{t}$ is the observation noise.

Let $x_{t}={\left[\begin{array}{cc} {SOC}_{t} & U_{1,t} \end{array}\right]}^{T}$, $u_{t}=I_{t}$, $y_{t}=U_{L,t}$. The standard form of the dynamic battery system’s description can be obtained as shown in Equation (25).

(25) $$\left\{\begin{array}{c} x_{t}=f\left(x_{t-1},I_{t}\right)+\varepsilon_{t}\\ y_{t}=g\left(x_{t},I_{t}\right)+\delta_{t} \end{array}\right.$$

### 3.2. Adaptive Unscented Kalman Filter

Assuming that the process noise and observation noise are uncorrelated white Gaussian noise, i.e., $\varepsilon_{t}\sim N(0,R_{t})$, $\delta_{t}\sim N(0,Q_{t})$, The standard UKF algorithm is as follows:

(1) State value and covariance initialization:

(26) $$\left\{\begin{array}{l} {\bar{x}}_{0}=E\left(x_{0}\right)\\ \Sigma_{0}=E[\left(x_{0}-{\bar{x}}_{0}\right){\left(x_{0}-{\bar{x}}_{0}\right)}^{T}] \end{array}\right.$$

(2) Sigma-point generation:

(27) $$\left\{\begin{array}{l} L_{t-1}^{\left[0\right]}=x_{t-1}\\ L_{t-1}^{\left[i\right]}=x_{t-1}+\gamma {\left(\sqrt{\Sigma_{t-1}}\right)}_{i},i=1,2,\ldots ,n\\ L_{t-1}^{\left[i+n\right]}=x_{t-1}-\gamma {\left(\sqrt{\Sigma_{t-1}}\right)}_{i},i=1,2,\ldots ,n \end{array}\right.$$

where $\gamma =\sqrt{\lambda +n}$, $\lambda =\alpha^{2}\left(n+k\right)-n$. The parameters α and k are used to determine how far all the sigma points are distributed from the mean value. In this research, α and k are set as 1 and 0, respectively.

(3) Coefficient calculation:

(28) $$\left\{\begin{array}{l} \omega_{m}^{\left[0\right]}=\frac{\lambda }{\lambda +n}\\ \omega_{c}^{\left[0\right]}=\frac{\lambda }{\lambda +n}+\left(1-\alpha^{2}+\beta \right)\\ \omega_{m}^{\left[i\right]}=\omega_{c}^{\left[i\right]}=\frac{1}{2\left(n+\lambda \right)},i=1,2,\ldots ,n \end{array}\right.$$

In fact, both $\omega_{m}^{\left[i\right]}$ and $\omega_{c}^{\left[i\right]}$ correspond to one sigma point, $L_{t-1}^{\left[i\right]}$, while $\omega_{m}^{\left[i\right]}$ is used for the mean value calculation and $\omega_{c}^{\left[i\right]}$ is used for the covariance calculation. Parameter β should be set as 2 for Gaussian distribution, which is the case in this investigation.

(4) Process update for sigma points and covariance:

(29) $${\bar{L}}_{t}^{*[i]}=f\left(L_{t-1}^{\left[i\right]},I_{t}\right)$$

(30) $${\bar{x}}_{t}=\sum_{i=0}^{2n}\omega_{m}^{\left[i\right]}{\bar{L}}_{t}^{*[i]}$$

(31) $${\bar{\Sigma }}_{t}=\sum_{i=0}^{2n}\omega_{c}^{\left[i\right]}\left({\bar{L}}_{t}^{*\left[i\right]}-{\bar{x}}_{t}\right){\left({\bar{L}}_{t}^{*\left[i\right]}-{\bar{x}}_{t}\right)}^{T}+R_{t}$$

(5) Kalman-gain calculation:

(32) $$\left\{\begin{array}{l} {\bar{L}}_{t}^{\left[0\right]}={\bar{x}}_{t}\\ {\bar{L}}_{t}^{\left[i\right]}={\bar{x}}_{t}+\gamma {\left(\sqrt{{\bar{\Sigma }}_{t}}\right)}_{i},i=1,2,\ldots ,n\\ {\bar{L}}_{t}^{\left[i+n\right]}={\bar{x}}_{t}-\gamma {\left(\sqrt{{\bar{\Sigma }}_{t}}\right)}_{i},i=1,2,\ldots ,n \end{array}\right.$$

(33) $${\bar{y}}_{t}^{\left[i\right]}=g({\bar{L}}_{t}^{\left[i\right]},I_{t})$$

(34) $${\hat{y}}_{t}=\sum_{i=0}^{2n}\omega_{m}^{\left[i\right]}{\bar{y}}_{t}^{\left[i\right]}$$

(35) $$S_{t}=\sum_{i=0}^{2n}\omega_{c}^{\left[i\right]}({\bar{y}}_{t}^{\left[i\right]}-{\hat{y}}_{t}){\left({\bar{y}}_{t}^{\left[i\right]}-{\hat{y}}_{t}\right)}^{T}+Q_{t}$$

(36) $${\bar{\Sigma }}_{t}^{x,y}=\sum_{i=0}^{2n}\omega_{c}^{\left[i\right]}({\bar{L}}_{t}^{\left[i\right]}-{\bar{x}}_{t}){\left({\bar{L}}_{t}^{\left[i\right]}-{\bar{x}}_{t}\right)}^{T}$$

(37) $$K_{t}={\bar{\Sigma }}_{t}^{x,y}S_{t}^{-1}$$

(6) State and covariance update:

(38) $$e_{t}=z_{t}-{\hat{y}}_{t}$$

(39) $$x_{t}={\bar{x}}_{t}+K_{t}e_{t}$$

(40) $$\Sigma_{t}={\bar{\Sigma }}_{t}-K_{t}S_{t}K_{t}^{T}$$

(7) Adaptive noise estimation:

(41) $$D_{t}=e_{t}{e_{t}}^{T}$$

(42) $$R_{t+1}=(1-d_{t})R_{t}+d_{t}K_{t}D_{t}K_{t}^{T}$$

(43) $$Q_{t+1}=\left(1-d_{t}\right)Q_{t}+d_{t}\left(\sum_{i=0}^{2n}\omega_{c}^{\left[i\right]}({\bar{y}}_{t}^{\left[i\right]}-{\hat{y}}_{t}){\left({\bar{y}}_{t}^{\left[i\right]}-{\hat{y}}_{t}\right)}^{T}+D_{t}\right)$$

where $d_{t}$ is expressed as

(44) $$d_{t}=\frac{1-b}{1-b^{t+1}},\left(0<b<1\right)$$

where b is the forgetting factor and was set as 0.95 in this study.

## 4. Experiments, Results, and Discussion

**Figure 10:**
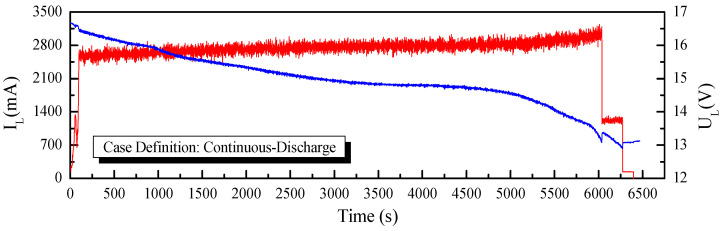
Current and voltage curves for continuous-discharge case.

**Figure 11:**
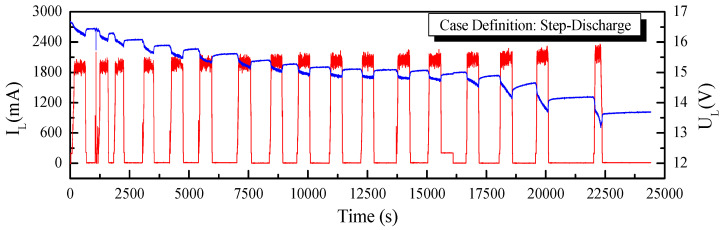
Current and voltage curves for step-discharge case.

To establish the performance of the application of the fast-OCV in the SOC estimation, two working conditions, continuous discharge and step discharge, as shown in Figure 10 and Figure 11, were tested. Furthermore, UKF and AUKF algorithms were adopted for the comparative investigations.

**Figure 12:**
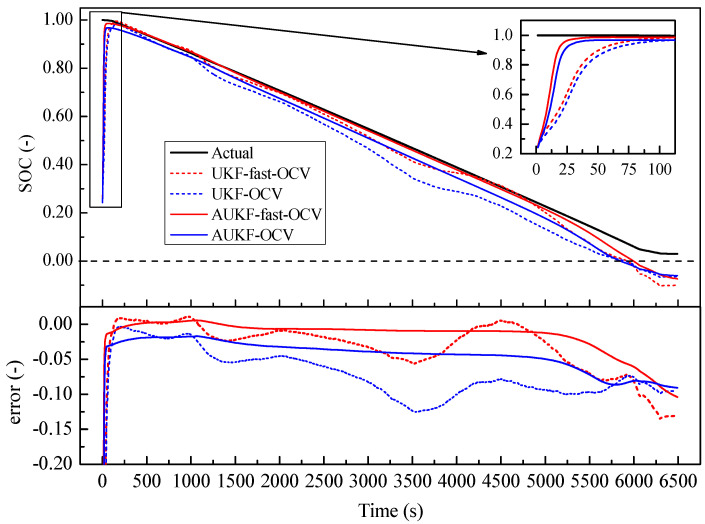
Comparison of SOC estimation: fast OCV versus traditional OCV under continuous-discharge condition.

**Figure 13:**
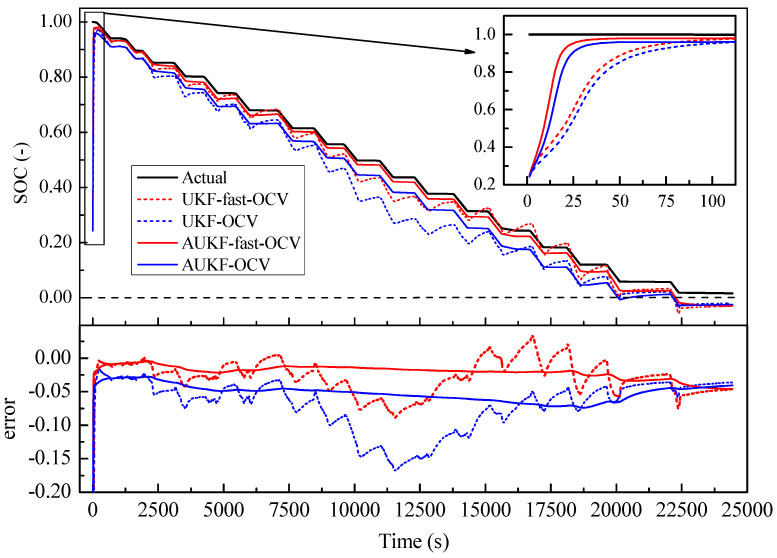
Comparison of SOC estimations: fast OCV versus traditional OCV under step-discharge conditions.

The initial value ${SOC}_{0}$ was set as 0.2, which was obviously not the real value, to establish how quickly each case converged with the real value. As shown in Figure 12 and Figure 13, all the predictions followed the trend of the SOC variation. However, we found that the fast OCV showed an obviously better accuracy. Furthermore, the fast OCV cases also showed faster convergence. This might have been due to the fact that the fast OCV was obtained under real application conditions, which are closer to actual working conditions than traditional ways of obtaining the OCV. However, all the cases lost track with the actual SOC when the battery was about to become empty. This phenomenon would possibly be ascribed to the drawback that the Thevenin model does not describe the diffusion effect, or to the fact that there was a lack of optimization on the current trace when carrying out the parameter identification.

According to the results shown in Figure 12 and Figure 13, it was also found that the AUKF showed a much better performance than the UKF. For the classical UKF, the covariance for the process noise and observation noise might not be optimal. However, with the adaptive estimation of the noise covariance, the Kalman filter worked more closely to the optimum performance; hence, the AUKF showed much better performances. It was also found in both cases that relatively large errors resulted when the SOC was low. This was mainly due to the fact that the OCV curve was obtained based on the Rint model. However, in the low-SOC situation, a distinct relaxation effect was observed, which was confirmed by the relatively large $R_{1}$ and $C_{1}$ values shown in Figure 9. Nevertheless, good accuracy in the SOC estimation was achieved within a large SOC span by using the fast-OCV curve with the AUKF algorithm.

All the results showed that the fast OCV exhibited very good performances when used for SOC estimation. These results seem to be unreasonable, as we observed that he fast OCV obviously deviated from the measured value, as shown in Figure 6. However, the ECM here was largely simplified, meaning that it may not have been able to describe the battery’s behavior in significant detail; thus, the OCV curve was not necessarily very close to the real value, but it was more compatible with the model [25]. In our case, the battery was described with the Thevenin model. The OCV was obtained with the assumption of the Rint model, which is of similar simplicity to the Thevenin model; hence, good compatibility was achieved between the OCV curve and the ECM. However, there are still some deficiencies in this method, specifically when the SOC is low, which suggests that poor compatibility between the OCV and ECM might be encountered. Therefore, more thorough work is still required in future investigations. When carrying out the parameter estimations with the limited-memory least-square algorithm, the batch mode showed much weaker noise. Considering the Kalman filters in this work, the performance in the parameter estimation might be further improved in further investigations in the future.

## 5. Conclusions

In this paper, the fast-OCV method was used to obtain the open-circuit voltage of a battery. By using Kalman filters, the battery SOC was estimated. To obtain the battery parameters, the dynamic battery model was discretized to form a linear equation for parameter solution by using the sampled terminal current and voltage data. To minimized data noise, a “batch mode” method was introduced to sufficiently use all the sampled data obtained with high frequency. Moreover, the limited-memory recursive least-square-algorithm was used to smooth the solution. The main conclusions of this research are as follows:

(1) The “batch-mode” proposed was able to make good use of all the data sampled, with a much higher frequency than the parameter-updating task, and it demonstrated a good de-noising effect in the battery-parameter estimation. Combined with the limited-memory recursive least-square algorithm, successful parameter estimation was achieved.
(2) The fast-OCV curve based on the Rint model was applied effectively for battery-SOC estimation. Compared with the traditionally obtained OCV curve, it even proved to offer much better accuracy in SOC estimation. Combined with an adaptive UKF, good accuracy in SOC estimation was achieved.
(3) Compared with the traditional approach to OCV curve identification, the fast-OCV method is much more time-efficient, with a completely fluctuating charging-and-discharging process. As hybrid power for UAVs should be configured with low capacity (light batteries), the fast-OCV method is highly suitable for these situations.

Nevertheless, the results also showed the relatively poor accuracy of the proposed method in SOC estimation in low-SOC situations. This was probably mainly be due to the higher relaxation effect when the SOC was low, as indicated by the relatively high $R_{1}$ and $C_{1}$ values. To perfect the OCV-curve identification, a first- or second-order ECM may need to be considered. Furthermore, the approach to fluctuating the current load on the battery may also affect the results of OCV curve obtained. The investigation of this topic would involve standard waves, like sinusoid waves and rectangular waves, or even standard battery-testing cycles. All these points are worthy of future investigations.

## Figures and Tables

**Table 1:** Key parameters of current transducer LA 55-P/SP1.

$\mathrm{Primary}\;\mathrm{Nominal}\;\mathrm{RMS}\;\mathrm{Current}\;(I_{p,N}$)	Primary Current $\mathrm{Measuring}\;\mathrm{Range}\;(I_{p,M}$)	Turns Ratio $(N_{p}/N_{s}$)	Supply Voltage $(\pm 5\%)\;(U_{C}$)
50 A	0~±100 A	1:2000	±12~15 V

**Table 2:** Measurement-resistance requirements of current transducer LA 55-P/SP1.

$\mathrm{Measuring}\;\mathrm{Resistance}\;(R_{M}$)				
Power Voltage	@TA = 70 °C		@TA = 85 °C	
	$R_{M}$ Min	$R_{M}$ Max	$R_{M}$ Min	$R_{M}$ Max
with ±12 V	0 Ω	215 Ω	0 Ω	210 Ω
	0 Ω	35 Ω	0 Ω	30 Ω
with ±15 V	0 Ω	335 Ω	30 Ω	330 Ω
	0 Ω	95 Ω	30 Ω	90 Ω

**Table 3:** Accuracy of parameters values of current transducer LA 55-P/SP1.

$\mathrm{Error}\;@I_{p},T_{A}=25\;{}^{\circ}C$		Linearity Error	$\mathrm{Offset}\;\mathrm{Current}\;@I_{p}=0,T_{A}=25\;{}^{\circ}C$	$\mathrm{Delay}\;\mathrm{Time}\;@10\%\;of\;I_{p,N}$	$\mathrm{Delay}\;\mathrm{Time}\;\mathrm{to}\;90\%\;\mathrm{of}\;I_{p,N}$ ^(1)^
@±15 V (±5%)	@±12~15 V (±5%)	<0.15%	within ±0.10 mA	<500 ns	<1 μs
±0.65%	±0.90%				

(1) For a di/dt = 100 A/μs.

**Table 4:** Battery information and coefficients of $R_{0}$ and OCV polynomials.

Coefficient	Value	Coefficient	Value	Coefficient	Value
$a_{0}$	0.18178	$a_{1}$	−0.92811	$a_{2}$	3.6568
$a_{3}$	−6.8357	$a_{4}$	5.9269	$a_{5}$	−1.7057
$a_{6}$	−0.22173	$b_{0}$	13.951	$b_{1}$	8.3961
$b_{2}$	−18.459	$b_{3}$	−4.6272	$b_{4}$	69.866
$b_{5}$	−82.283	$b_{6}$	29.877		
Battery information: Lipo, 4S1P, 14.8V,45C					

## Data Availability

Not applicable.
